# Predicting Oral HIV Pre‐Exposure Prophylaxis Efficacy in Cisgender Women Across Pregnancy and Postpartum

**DOI:** 10.1002/jcph.70278

**Published:** 2026-09-03

**Authors:** Yifan Yu, Kristina M. Brooks, Gustavo F. Doncel, Brookie M. Best, Mark A. Marzinke, Mark Mirochnick, Peter L. Anderson, Landon Myer, Connie Celum, Renee Heffron, Jenell Coleman, Dvora L. Joseph Davey, Lanxin Zhang, Max von Kleist, Craig W. Hendrix, Jeremiah D. Momper, Robert Bies, Rachel K. Scott

**Affiliations:** ^1^ School of Pharmacy and Pharmaceutical Sciences University at Buffalo Buffalo NY USA; ^2^ Skaggs School of Pharmacy and Pharmaceutical Sciences University of Colorado‐Anschutz Medical Campus Aurora CO USA; ^3^ CONRAD Eastern Virginia Medical School Old Dominion University Norfolk VA USA; ^4^ Skaggs School of Pharmacy and Pharmaceutical Sciences University of California San Diego La Jolla CA USA; ^5^ Pediatrics Department University of California San Diego School of Medicine‐Rady Children's Hospital San Diego San Diego CA USA; ^6^ School of Medicine Johns Hopkins University Baltimore MD USA; ^7^ Chobanian & Avedisian School of Medicine Boston University Boston MA USA; ^8^ Division of Epidemiology and Biostatistics School of Public Health University of Cape Town Cape Town South Africa; ^9^ Departments of Global Health Medicine & Epidemiology miology University of Washington Seattle WA USA; ^10^ Department of Medicine University of Alabama at Birmingham Birmingham AL USA; ^11^ Division of Infectious Diseases Geffen School of Medicine University of California Los Angeles Los Angeles CA USA; ^12^ Project Group 5 “Systems Medicine of Infectious Disease” Robert‐Koch Institute Berlin Germany; ^13^ Medstar Health Washington DC USA; ^14^ Georgetown University School of Medicine Washington DC USA

**Keywords:** antiretroviral, HIV, population pharmacokinetics, pregnancy, quantitative pharmacology, trial simulation

## Abstract

Cisgender women face an increased risk of HIV acquisition during pregnancy and postpartum. Oral pre‐exposure prophylaxis (PrEP) with emtricitabine and tenofovir disoproxil fumarate (F/TDF) is recommended in pregnancy despite limited published pharmacokinetic (PK) and efficacy data during pregnancy. Altered PK during pregnancy may reduce prophylactic efficacy and necessitate higher adherence. Tenofovir alafenamide (TAF) is a newer tenofovir prodrug with higher tenofovir diphosphate (TFV‐DP) exposure in peripheral blood mononuclear cells (PBMCs), but the prophylactic efficacy of F/TAF across different reproductive stages in women is understudied. We adapted a multiscale modeling framework to predict the prophylactic efficacy of F/TDF and F/TAF across varying adherence levels and during the second trimester, third trimester, and 6–12 weeks postpartum. Clinical trial simulations were conducted utilizing PBMC TFV‐DP and emtricitabine triphosphate concentrations as surrogate markers for F/TAF and F/TDF efficacy. For both F/TDF and F/TAF, prophylactic efficacy was lowest during the second trimester, increased in the third trimester (but remained below nonpregnant levels), and was highest postpartum. At high adherence levels of five or more pills per week, predicted effectiveness remained high across pregnancy and postpartum and effectiveness exceeded 97% for both regimens, with maximum decreases in the second trimester of only 0.9 percent point for F/TDF and 0.3 percent point for F/TAF. These findings suggest that adherence is the primary determinant of PrEP effectiveness during pregnancy and postpartum, while pregnancy‐related PK changes have a limited impact on efficacy.

## Introduction

Globally, cisgender women and individuals assigned female at birth (subsequently “women”) and girls bear a disproportionate burden of new HIV infections. Vulnerability in this population increases during pregnancy and postpartum due to behavioral and physiological changes, elevating the risks of both maternal HIV acquisition and subsequent perinatal transmission.^1^, ^2^ Pre‐exposure prophylaxis (PrEP) is a key HIV prevention strategy; oral tenofovir disoproxil fumarate/emtricitabine (F/TDF) and long‐acting injectables cabotegravir (CAB LA) and lenacapavir (LEN) are currently approved for prevention in cisgender women with potential exposure to HIV. Although oral PrEP with F/TDF is highly effective, adherence is critical to achieve adequate protection against HIV infection. Anderson et al reported sex‐based differences in pharmacologic forgiveness, with men achieving similar protection at lower adherence levels, based on intraerythrocytic tenofovir diphosphate (TFV‐DP) data from the HPTN 083 and 084 trials.^3^ Marrazzo et al subsequently conducted a pooled analysis of 11 post‐approval studies of F/TDF and showed that it is highly effective in women who take more than four doses per week.^4^ Moore et al calibrated an adherence–efficacy curve based on intraerythrocytic TFV‐DP concentrations to the reduction in HIV incidence in women. The analysis suggested that two, four, and seven F/TDF tablets per week can reduce HIV incidence by 59.3%, 83.8%, and 95.5%, respectively.^5^ The World Health Organization (WHO), along with many national HIV guidelines, recommends the use of oral F/TDF in pregnant and postpartum populations due to the published safety profile and pharmacokinetic (PK) data.^6^ However, physiologic changes during pregnancy decrease drug exposure and potentially protective efficacy.

Tenofovir alafenamide (TAF), a prodrug of tenofovir, offers a distinct pharmacokinetic profile compared to TDF. TAF achieves up to a seven‐fold increase in intracellular TFV‐DP concentrations in HIV target cells, while simultaneously reducing plasma tenofovir concentrations by approximately 90%.^7^, ^8^ Although F/TAF is not currently approved by the United States Food and Drug Administration (FDA) for HIV prevention, updated guidance from the International Antiviral Society‐USA Panel recommends F/TAF for prevention of HIV acquisition from vaginal exposure.^9^ Although TAF exposure also declines during pregnancy,^10^, ^11^, ^12^ our prior PK modeling suggests that it may maintain significantly higher intracellular TFV‐DP levels in PBMC than TDF during the pregnant and postpartum periods, potentially providing greater protection.^13^

Mathematical modeling and simulation have been used to investigate the effectiveness of PrEP across different populations and anatomical exposure sites.^14^, ^15^ Duwal et al established a multiscale systems‐pharmacology workflow to assess the prophylactic efficacy of different nucleotide reverse transcriptase inhibitors.^16^ Zhang et al employed this workflow to conduct bottom‐up modeling strategies evaluating F/TDF efficacy in women, estimating the prophylactic efficacy across different adherence scenarios and target tissues.^17^ Their results suggest that adherence, rather than biological sex differences, is the more critical factor influencing PrEP effectiveness. However, limited research has evaluated PrEP efficacy during pregnancy and the postpartum and the relative impact of adherence under pregnancy‐associated PK change during these periods.

Building upon these methodologies, we used a model‐based framework to investigate the prophylactic efficacy of F/TDF and F/TAF in women across pregnancy and the postpartum period. Through simulating the prophylactic efficacy across various adherence patterns and conducting clinical trial simulations, this study aimed to (1) compare their predicted efficacy in this high‐risk population and (2) validate PBMCs as an appropriate surrogate target site for both regimens.

## Methods

This study is a secondary analysis of deidentified data from previously published studies and all data were deidentified prior to data sharing and this analysis. The University at Buffalo reviewed this study (institutional review board ID: STUDY00006180) and determined that as this research does not involve human subjects, institutional review board approval was not required.

The model‐based framework we used in this analysis was based on previous work by Duwal et al and Zhang et al.^16^, ^18^ We made modifications to adapt the framework to pregnant and postpartum women, including physiologically related PK changes, stochastic adherence patterns, and increased probability of HIV acquisition during pregnancy and the postpartum period. The core assumptions of the original framework were preserved, and further details are provided below.

### Surrogate Site Justification

The analysis was under the hypothesis that concentrations in PBMC are the primary surrogate for PrEP protective efficacy. Zhang et al previously reanalyzed data from major F/TDF PrEP clinical trials inclusive of female participants and compared simulated HIV incidence derived from observed clinical data with model‐predicted incidence. They found that assuming the female genital tract as the target site significantly underestimated prophylactic efficacy.^17^ Moreover, cervical, vaginal, and rectal biopsies are generally infeasible during pregnancy, and the inherent variability in tissue PK data further limits the ability to accurately predict local drug exposure in these compartments during pregnancy. Given these considerations, we used intracellular TFV‐DP and emtricitabine triphosphate (FTC‐TP) concentrations in PBMCs to drive the prophylactic efficacy.

### Pharmacokinetics of TDF, TAF, and FTC

We simulated individual adherence profile by generating a binary vector representing each virtual female participant's daily medication‐taking behavior. Specifically, for each dosing day, a binary outcome (1 indicating a dose taken and 0 indicating a missed dose) was sampled from a Bernoulli distribution

$$d_{i}\;∼\;\mathrm{Bernoulli}\;\left(\frac{\mathrm{Adherence}\;\mathrm{level}}{7\;\mathrm{doses}\;\mathrm{per}\;\mathrm{week}}\right),\;i\;=1,\;2,\;\text{…}.,\;T$$

where $d_{i}$ represents the binary outcome on day i, T is the total number of dosing days. Adherence level is integer between 1 and 7 indicating the number of doses taken per week. Individuals were assumed to remain at fixed adherence level throughout the sampling.

Based on the adherence profiles, we simulated PBMC TFV‐DP PK profiles following TAF and TDF administration across reproductive stages in women, including nonpregnant, second trimester, third trimester, and 6–12 weeks postpartum populations, for 1000 virtual participants. These simulations were performed using our previously developed semi‐mechanistic population PK (popPK) model^19^ and mrgsolve package in R.

For FTC, two clinical studies have evaluated FTC‐TP concentrations in PBMC during pregnancy and postpartum,^20^, ^21^ but no PK model has been developed to date to describe these changes. In this case, we first developed an FTC popPK model in nonpregnant women without HIV based on the plasma FTC and PBMC FTC‐TP data from the CONRAD 137 study^8^ and then combined the developed model with the identified covariates between pregnancy trimester and FTC apparent clearance reported by Valade et al where we assume FTC apparent clearance increases by 18% during the second and the third trimester and returns to the nonpregnant value after delivery.^22^ FTC PK profiles were subsequently simulated based on the generated adherence profiles for 1000 virtual participants.

### Pharmacodynamics of F/TDF and F/TAF

The molecular mechanism of action (MMOA) model by Iannuzzi and von Kleist described the concentration–efficacy relationship of F/TDF.^23^ This MMOA model incorporated the depletion effect on the endogenous dNTP pool and captured the synergistic contributions of F/TDF observed in vitro. The potential mechanism of this depletion effect might be attributed to the TFV‐mediated upregulation of 5′‐nucleotidase.^24^ Based on this assumption, F/TAF is expected to show a similar or enhanced depletion effect on the dNTP pool as F/TDF due to the higher PBMC loading effect. Based on these assumptions, the combined effects of intracellular TFV‐DP and FTC‐TP were pre‐simulated using the MMOA model, following the approach described by Zhang et al.^17^

### Modeling Virus Exposure During Pregnancy and Postpartum

To calculate the number of transmitted viruses that can reach the surrogate site and start productive infection (inoculum size), we employed the method developed by Duwal et al.^16^ In this framework, the inoculum size n is modeled as a binomially distributed random variable:

$$n\;∼\;\mathrm{Binomial}\left(∥VL^{m}∥,\;r\right)$$

where VL represents the viral load in the donor, m represents the power exponent, ∥·∥ denotes the next integer function, and r represents the success rate, that is, the probability at which a single HIV virion can travel from the site of exposure to the surrogate site and initiate a productive infection. Using this model, the mean probability of HIV acquisition per condomless sex act with a partner having a viral load of 10,000 copies/mL (in the absence of any PrEP agent) is computed as:

$$P\;\left(\inf|\mathrm{VL}\;=10,000\;\mathrm{copies}/\mathrm{mL}\right)=\sum_{n=0}^{∥10,000\;\mathrm{copies}/mL^{m}∥}\left(\begin{array}{c} ∥10,000\;\mathrm{copies}/mL^{m}∥\\ n \end{array}\right)\cdot r^{n}\cdot {\left(1-r\right)}^{\left(∥10,000\;\mathrm{copies}/mL^{m}∥-n\right)}\cdot \left(1-{\left(1-\alpha \right)}^{n}\right)$$

where n represents the inoculum size, α represents the HIV acquisition probability when inoculum size equals to 1 virus.

Thomson et al analyzed data from 2751 HIV‐serodiscordant couples in the Partners PrEP study and reported HIV acquisition probabilities per sex act among women across different reproductive stages.^2^ These estimates were adjusted for a partner viral load of 10,000 copies/mL and without the use of PrEP. In our analysis, we attribute the observed increases in acquisition probability during certain reproductive stages to a biological increase in the success rate r. This elevated r leads to a higher expected inoculum size and, consequently, an increased risk of infection.

Based on this assumption, we inferred stage‐specific values of r and use them to estimate the corresponding inoculum size distributions and HIV acquisition probabilities.

### Prophylactic Efficacy Prediction

We adopted the probability generating system developed by Zhang et al^18^ to predict the long‐term prophylactic efficacy of F/TDF and F/TAF in women during the pregnant and postpartum periods. The system is based on a continuous–time discrete state Markov chain that models HIV viral replication. The extinction probability over time is calculated by solving a set of ordinary differential equations (ODEs), where PBMC TFV‐DP and FTC‐TP concentrations modulate ODE parameters. Details of the viral dynamic model and probability generating system are described in Zhang et al.^18^

For each adherence and reproductive stage scenario, we simulated the extinction probability over 84 days from the initiation of PrEP for each of the 1000 virtual individuals. To reflect the system's “steady state,” we excluded both the initial and final 28 days of the simulation period and analyzed only the intermediate 28 days for each scenario.

To account for the stochastics in viral exposure, the distribution of inoculum size was used to calculate probability of extinction using the equation below:


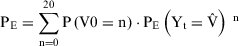




As the distribution across all reproductive stages are highly skewed toward 0, we only consider the inoculum size ranging from 0 to 20. The prophylactic efficacy, that is, the reduction of infection probability per challenge, was then calculated as below:

$$\varphi \;=1-\;\frac{P_{s}\left(\inf\right)}{P_{\emptyset }\left(\inf\right)}=\frac{P_{E,s}-P_{E,\emptyset }}{1-P_{E,\emptyset }}$$

where $P_{E,s}$ and $P_{E,\emptyset }$ represent extinction probability with and without antiretrovirals.

### Clinical Trial Simulation

To validate PBMCs as an appropriate surrogate target site, we performed Gillespie simulations to simulate the PURPOSE 1 study, currently the only clinical trial evaluating both F/TDF and F/TAF effectiveness in women. We adapted the stochastic model developed by Zhang et al below to simulate infection in the F/TAF and F/TDF arms in PURPOSE 1 study^17^:

$$R_{1}\;:\;S\overset{r_{\inf}}{\to }\;I\;R_{2}\;:\;S\overset{r_{\mathrm{drop}}}{\to }\;\emptyset$$

At baseline, state S enrolled a fixed number of individuals in each arm (2136 in F/TAF arm and 1068 in F/TDF arm). The rates $r_{\inf}$ and $r_{\mathrm{drop}}$ represent the incidence rate and dropout rate, respectively. As for the $r_{\inf}$, we include both the background HIV incidence and time‐varying PrEP adherence:

$$\;r_{\inf}=r_{\inf-\mathrm{background}}\;\;\left(1-\hat{\varphi }\right)$$

where $r_{\inf-\mathrm{background}}$ is the background HIV incidence from the cross‐sectional incidence cohort. In this analysis, a gamma distribution was fitted to describe the reported median and 95% confidence interval values of the background incidence. During the simulation, $r_{\inf-\mathrm{background}}$ was randomly sampled from this gamma distribution. Based on the dichotomization method developed by Zhang et al, we also estimated the background incidences for the F/TAF and the F/TDF arm based on the observed number of infections in each arm.^17^ The workflow is described in the Supplemental Information. The term $\hat{\varphi }$ denotes the time‐averaged prophylactic efficacy predicted based on adherence, as estimated from DBS TFV‐DP concentrations:

$$\;\hat{\varphi }=\sum_{j=1}^{7}{\hat{\varphi }}_{j}P\left(\mathrm{adherence}=j\right)$$

where P(adherence=j) reflects the relative frequency of each adherence level from a randomly preselected 10% of participants in each group and is used as the empirical probability. For the three adherence categories (low, medium, and high) used in PURPOSE 1 trial, we assumed that the probability of taking any given number of pills within each category was equal, with the exception that participants in the low‐adherence group were assumed to take no drug. The dropout rate $r_{\mathrm{drop}}$ is estimated by subtracting the infection rate $r_{\inf}$ from the inverse of the average follow‐up time per individual. Here, the inverse of the average follow‐up time approximates the overall crude dropout rate, which includes all causes of dropout or loss to follow‐up observed in the study population during the follow‐up period.

Using these parameters, we simulated 1000 clinical trials (with each n = 2136 in F/TAF arm and n = 1068 in F/TDF arm) for both the F/TAF and F/TDF arms. To test our hypothesis, the simulated infection numbers in both arms were compared against the observed outcomes from the PURPOSE 1 study.

## Results

### Pharmacokinetic Models

**Figure 1:**
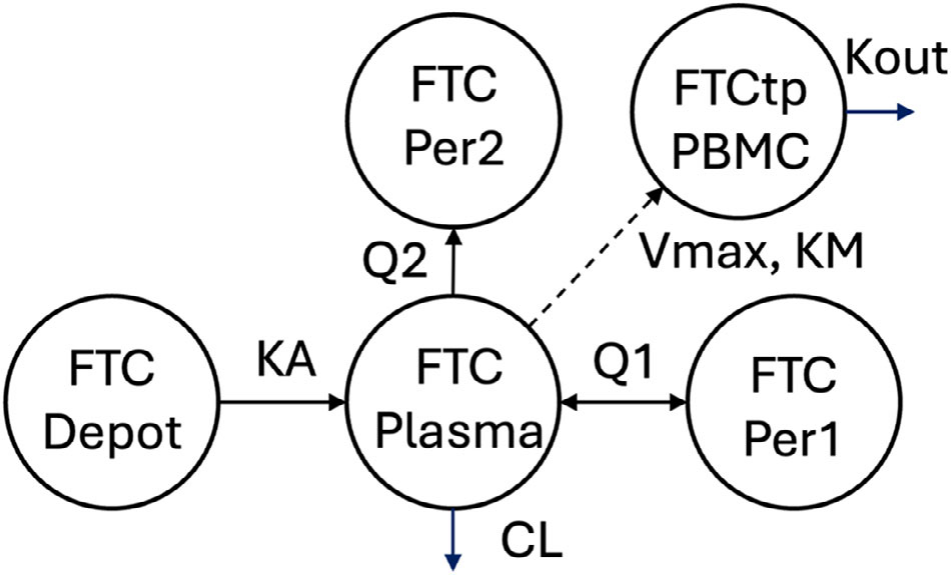
Model structure of FTC popPK model.

The model structure of TAF and TDF was previously reported. For FTC and its active anabolite, FTC‐TP, a three‐compartment model with a biophase compartment adequately describes the plasma FTC and PBMC FTC‐TP PK data observed in nonpregnant women without HIV from the CONRAD 137 study.^8^ A saturable formation kinetics of PBMC FTC‐TP using Michaelis–Menten equation and a linear elimination from PBMC was selected. The model structure is described in Figure 1, and the ODEs are provided in the Supplemental Information. The final estimates of PK parameters are shown in Table S1. The apparent half‐life of PBMC FTC‐TP calculated based on the simulated PK profiles was 29.4 h.

### Combined Effect of TFV‐DP and FTC‐TP

The simulated combined effect of TFV‐DP and FTC‐TP are shown in Figure S1. When depletion of the endogenous dNTP pool is incorporated into the model (Panel A), the combined inhibitory effect of TFV‐DP and FTC‐TP is enhanced compared to the scenario assuming independent action (Panel B).

### HIV Virus Exposure During Pregnancy and Postpartum

**Figure 2:**
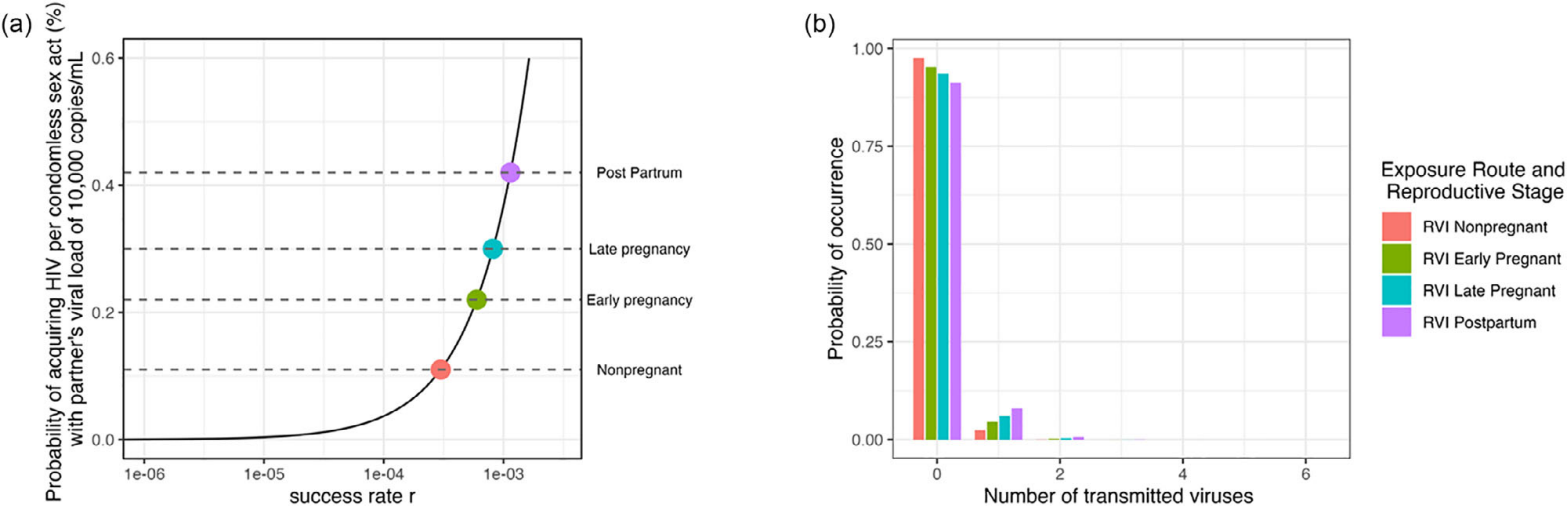
HIV viral exposure model during pregnancy and the postpartum period without any PrEP regimen. (a) Relationship between success rate r, the probability at which a single HIV virion can travel from the site of exposure to the target site and initiates a productive infection, and the corresponding adjusted infection probability per condomless sex act, assuming a partner viral load of 10,000 copies/mL. Colored dots represent reported HIV acquisition probabilities at different reproductive stages. (b) Estimated distribution of viral inoculum size across reproductive stages. We assumed this rightward shift from nonpregnant to postpartum period caused increased susceptibility.

Figure 2 illustrates the relationship between viral success rate and the adjusted probability of HIV acquisition per condomless sex act across different pregnancy stages, from nonpregnant/pre‐conception to postpartum. As shown in Panel A, the adjusted probability of infection increases progressively from the nonpregnant to the postpartum stage. The colored dots represent the estimated infection risk at each stage, corresponding to specific success rates r (nonpregnant: 0.000299, second and third trimester: 0.000815, postpartum period [from delivery to Week 24]: 0.001142).

Panel B shows the estimated distribution of viral inoculum size. The distributions are highly skewed toward 0 due to the overall low success rate. According to our hypothesis, the increasing success rate across reproductive stages leads to a slight rightward shift in the inoculum size distribution from the nonpregnant to the postpartum period.

### Prophylactic Efficacy Prediction During Different Reproductive Stages

**Figure 3:**
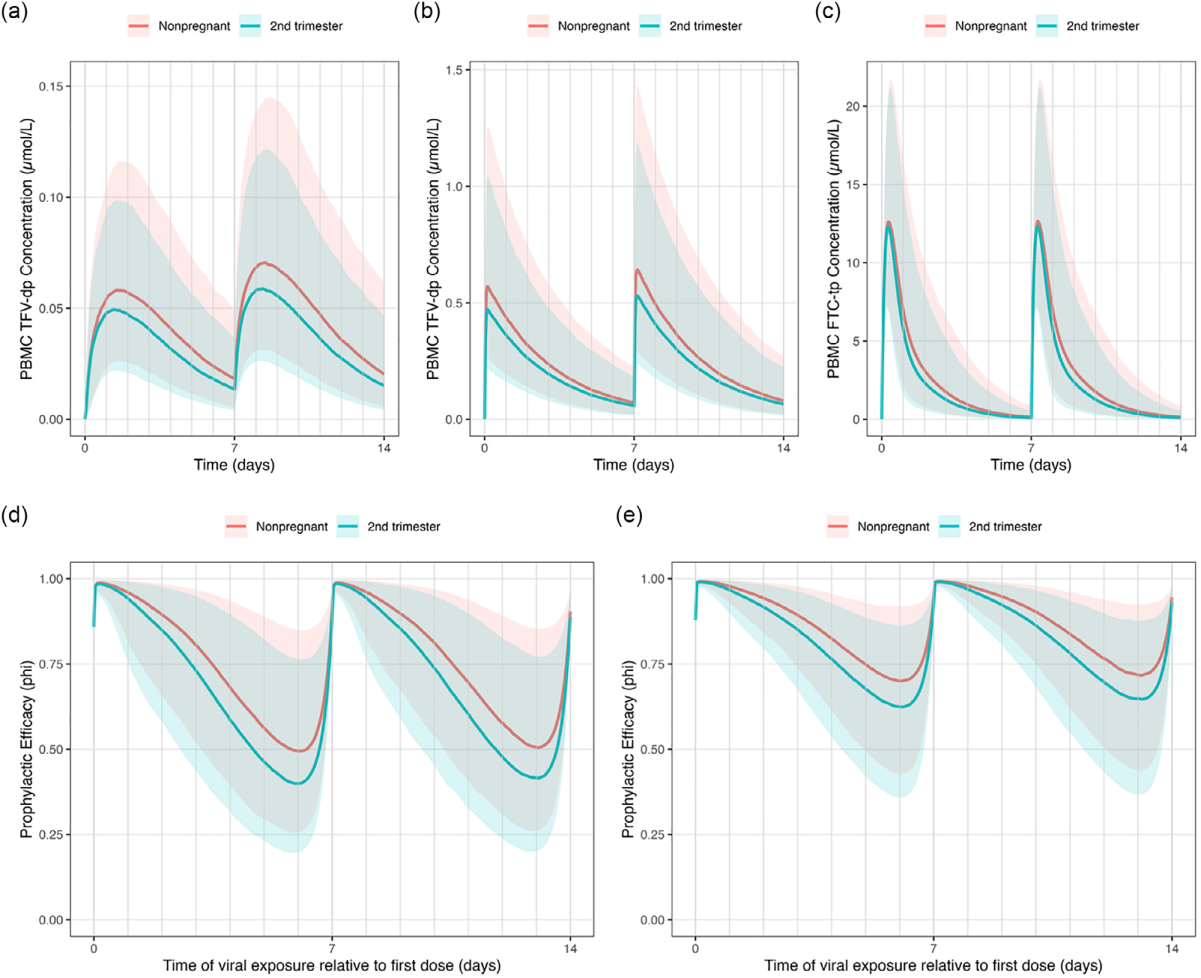
Example of prophylactic efficacy prediction in nonpregnant and pregnant women. Simulated PBMC TFV‐DP and FTC‐TP concentration profiles following one pill per week of F/TDF (a, c) or F/TAF (b) in nonpregnant and second trimester individuals, respectively. Predicted F/TDF (d) and F/TAF (e) prophylactic efficacy based on simulated PBMC PK profiles, the modeled combination effect incorporating dNTP depletion, estimated distributions of viral inoculum size during pregnancy and the second trimester, and the probability generating system.

Figure 3 displays an example of simulated intracellular TFV‐DP and FTC‐TP PK profiles and corresponding prophylactic efficacy profiles. We simulated TFV‐DP and FTC‐TP PK profiles for 1000 nonpregnant women and 1000 pregnant women in the second trimester, assuming a low adherence scenario of one pill per week (Panels A and B); the predicted prophylactic efficacy is shown in Panel C. The simulation demonstrates decreased predicted prophylactic efficacy due to reduced intracellular exposure of TFV‐DP and FTC‐TP during the second trimester.

**Figure 4:**
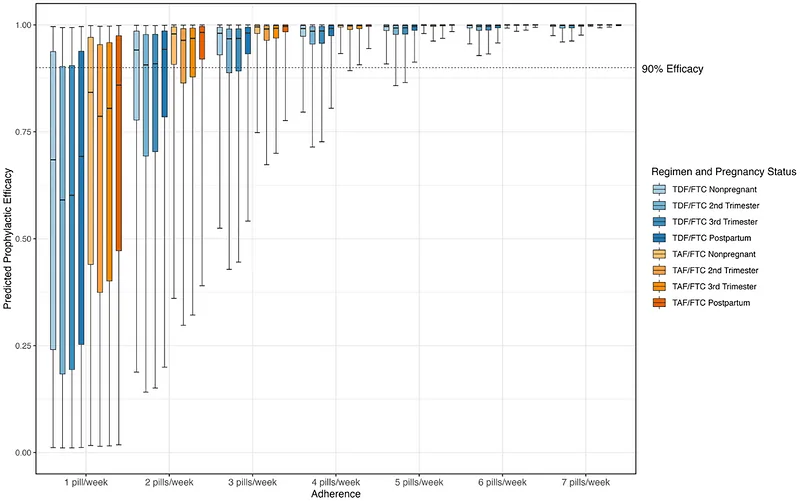
Predicted prophylactic efficacy for F/TAF and F/TDF with different adherence across different reproductive stages. Boxplot showed the median and IQR of simulated efficacy and the whiskers represents 2.5%–97.5% range. The back dashed line represents 90% efficacy.

Figure 4 summarizes the calculated prophylactic efficacy φ during the intermediate 28 days of the simulation period (672 time points) for each of the 1000 virtual individuals across different adherence and reproductive stages. The boxplot shows the median and IQR of simulated efficacy and the whiskers represent the 2.5%–97.5% range. The mean and 95% quantile range are shown in Table 1. A modest reduction in predicted effectiveness was observed for both F/TDF and F/TAF during the second and third trimesters, particularly at lower adherence levels. Assuming PBMCs are the surrogate target site of TFV‐based PrEP efficacy, F/TAF demonstrated higher efficacy than F/TDF across all scenarios, consistent with higher PBMC TFV‐DP exposure after F/TAF administration.

**Table 1:** Predicted Prophylactic Efficacy Across Different Adherence and Reproductive Stages

Adherence	F/TAF^a^ Average Predicted Prophylactic Efficacy (95% Quantile Range)				F/TDF^b^ Average Predicted Prophylactic Efficacy (95% Quantile Range)			
	Nonpregnant	2nd Trimester	3rd Trimester	Postpartum	Nonpregnant	2nd Trimester	3rd Trimester	Postpartum
1 pill/week	69.2% (1.7%, 99.8%)	65.6% (1.5%, 99.7%)	66.9% (1.5%, 99.7%)	70.6% (1.8%, 99.9%)	59.2% (1.2%, 99.6%)	54.5% (1.1%, 99.4%)	55.1% (1.1%, 99.4%)	59.7% (1.2%, 99.6%)
2 pills/week	90.8% (36.1%, 100.0%)	88.3% (29.8%, 100.0%)	89.1% (32.2%, 100.0%)	91.6% (39.0%, 100.0%)	83.7% (18.8%, 99.9%)	79.6% (14.1%, 99.8%)	80.2% (15.1%, 99.8%)	84.1% (20.0%, 99.9%)
3 pills/week	97.1% (74.8%, 100.0%)	95.8% (67.3%, 100.0%)	96.2% (70.0%, 100.0%)	97.5% (77.6%, 100.0%)	93.0% (52.5%, 100.0%)	90.4% (42.8%, 100.0%)	90.8% (44.5%, 100.0%)	93.3% (54.1%, 100.0%)
4 pills/week	99.2% (93.3%, 100.0%)	98.6% (89.3%, 100.0%)	98.8% (90.7%, 100.0%)	99.3% (94.5%, 100.0%)	97.1% (79.6%, 100.0%)	95.6% (71.4%, 100.0%)	95.8% (72.7%, 100.0%)	97.2% (80.5%, 100.0%)
5 pills/week	99.8% (98.0%, 100.0%)	99.5% (96.2%, 100.0%)	99.6% (96.9%, 100.0%)	99.8% (98.4%, 100.0%)	98.7% (90.9%, 100.0%)	97.8% (85.8%, 100.0%)	97.9% (86.5%, 100.0%)	98.7% (91.3%, 100.0%)
6 pills/week	99.9% (99.2%, 100.0%)	99.8% (98.5%, 100.0%)	99.9% (98.8%, 100.0%)	99.9% (99.5%, 100.0%)	99.3% (95.6%, 100.0%)	98.8% (92.8%, 100.0%)	98.9% (93.2%, 100.0%)	99.4% (95.8%, 100.0%)
7 pills/week	100.0% (99.7%, 100.0%)	99.9% (99.3%, 100.0%)	100.0% (99.5%, 100.0%)	100.0% (99.8%, 100.0%)	99.6% (97.5%, 100.0%)	99.4% (96.0%, 100.0%)	99.4% (96.2%, 100.0%)	99.7% (97.6%, 100.0%)

^a^ F/TAF: FTC 200 mg and TAF 25 mg per tablet.

^b^ F/TDF: FTC 200 mg and TDF 300 mg per tablet.

### Clinical Trial Simulation

We conducted 1000 stochastic simulations for both the F/TAF and F/TDF PrEP options to predict HIV infection outcomes in nonpregnant women in a virtual trial mimicking the PURPOSE 1 study. The distribution of simulated background HIV incidences are shown in Figure S2. The average prophylactic efficacy in the PURPOSE 1 trial was estimated based on the predicted adherence levels, shown in Figure S3.

We applied the background incidence derived from the cross‐sectional incidence cohort. In the F/TAF arm, the cumulative number of simulated infections over time is presented in Figure S4A. Across simulations, there were a median of 27 infections and a 95% quantile range of 15 to 40; the distribution is illustrated in Figure S4B. The observed number of infections in the PURPOSE 1 F/TAF arm was 39, which is at the upper end of this range. In contrast, for the F/TDF arm, the time course of cumulative infections is shown in Figure S4C. We found a median of 17 infections and a 95% quantile range of 9 to 28, as depicted in Figure S4D. The observed number of infections in the PURPOSE 1 F/TDF arm was 16, which falls within our predicted range. The simulation results based on the background incidence estimated using the dichotomization method are shown in Figure S5. Incorporating the arm‐specific HIV incidence, our simulations may better reproduce the number of infections in both study arms.

## Discussion

This study presents the first modeling and simulation analysis to evaluate the effectiveness of oral PrEP in pregnant and postpartum women. While previous studies have characterized the PK changes of F/TDF and F/TAF in pregnant and postpartum women living with HIV, their implications for PrEP efficacy have not been quantitatively explored. Our analysis incorporated PK of both TAF and TDF, allowing a direct comparison of their predicted effectiveness based on our model assumptions, and evaluation of PBMC concentrations as a surrogate marker for PrEP effectiveness in women.

For TDF, increased glomerular filtration rate (GFR) during pregnancy leads to higher TFV clearance and lower systemic exposure.^25^, ^26^, ^27^, ^28^ Brooks et al reported a significant association between plasma TFV monoester and TFV‐DP concentrations in PBMCs and DBS, suggesting that TFV monoester may be a chemical moiety responsible for facilitating cell loading.^29^ The model predicted a reduction in the PBMC TFV‐DP concentration based on the assumption that plasma TFV is the only chemical molecule actively targeted to the PBMC compartment, due the absence of TFV monoester in our model. In contrast to TDF, the mechanisms contributing to altered plasma TAF concentrations during pregnancy and postpartum are not fully understood.^10^, ^11^ Potential factors include increased plasma volume and changes in bioavailability. FTC's apparent clearance is also impacted by increased GFR, leading to a reduction in plasma FTC concentration.^22^, ^30^ However, due to the relatively small increase in apparent clearance and nonlinear formation of PBMC FTC‐TP from plasma FTC, our model predicted less impact on PBMC FTC‐TP concentrations.

Using PBMC concentrations as a surrogate marker, although TAF leads to significantly higher intracellular concentrations than TDF, the difference in prophylactic efficacy is minimal at moderate to high adherence. As shown in Figure 4, both regimens hit an efficacy ceiling near 100% at these levels. The efficacy difference is more significant when adherence drops to 1 pill/week. However, significant variability was observed in the lower adherence level for both F/TDF and F/TAF, making interpretation difficult. For both regimens, median efficacy was lower during the second and third trimesters and higher during the postpartum period compared to the nonpregnant state at each adherence level, which aligns with the previously modeled PK during pregnancy and postpartum period. Notably, adherence and the choice of regimen were the main drivers of prophylactic efficacy regardless of pregnancy stage. For F/TDF, our results suggest that pregnancy has minimal impact at higher adherence levels, supporting the WHO recommendations for the utilization F/TDF for HIV prevention during pregnancy and postpartum.

PURPOSE 1 trial also highlighted adherence as a key determinant of F/TAF effectiveness. Among 39 HIV new infections that occurred in the F/TAF arm, nearly all were attributable to low adherence.^31^ Subgroup analyses revealed that F/TAF was efficacious in participants with moderate to high adherence. Based on the analysis by Bekker et al, taking two or more F/TAF doses per week can reduce the odds of HIV acquisition for women by 89% (51% to 99%). While the corresponding estimate for F/TDF is not provided, the odds of HIV acquisition is not different comparing F/TAF to F/TDF. In addition, the crude percentage of participants with incident HIV infections with low or no adherence (which they define as <2% per week) within oral PrEP arm are nearly the same: F/TAF 92% (34/37) and F/TDF 93% (13/14). No incidence data are presented for discrete adherence bands typically used in prior oral PrEP adherence assessments, namely 7 and 4–6 doses per week.

However, our simulation results suggest a potential disconnect between observed clinical trial outcomes and model‐predicted effectiveness. Our mechanistic model assumed that only PBMC concentrations were predictive of PrEP efficacy and tissue PK had no additive value; therefore, the high TFV‐DP concentrations at more than two pills per week of F/TAF dosing should lead to near complete protection among women in our clinical trial simulation. This assumption, however, led to an underprediction of HIV infections in the simulated F/TAF arm when compared to the reported PURPOSE 1 trial we simulated. Predictions of F/TAF efficacy based on PBMC FTC‐TP and TFV‐DP concentrations were sensitive to assumptions regarding adherence stratification, and differences in predicted efficacy may be explained by how low adherence levels are interpreted in clinical trial data. Besides, TAF and TDF exhibit very different PK in local mucosal tissues, with TAF achieving lower TFV‐DP concentrations in the female genital tract and rectal tissues compared with TDF.^32^ Potential differences in tissue exposure may also contribute to the discrepancy in the predicted F/TAF effectiveness. In contrast, the efficacy of F/TDF by the same mechanistic model in our clinical trial simulation predicted results that corresponded reasonably well to PURPOSE 1 results. These findings suggest that model prediction performance across two regimens requires further validation using additional clinical data.

Our findings emphasize the importance of adherence to F/TDF during pregnancy and postpartum. Joseph Davey et al reported suboptimal F/TDF adherence during the peripartum period, with further decline postpartum,^33^ highlighting the challenge of maintaining adherence during and after pregnancy. Our model‐predicted prophylactic efficacy across adherence levels indicates that the impact of the physiologic changes of pregnancy is more significant at lower adherence levels, underscoring the importance of high adherence to oral PrEP in pregnancy to ensure adequate protection.

While dosing forgiveness is well established in men who have sex with men (MSM) and transgender women, the minimum adherence threshold required for effective protection in cisgender women remains unclear. Moore et al highlighted substantial uncertainty and variability in adherence thresholds.^34^ Compared to Moore's analysis, Zhang et al's modeling tended to predict higher effectiveness of F/TDF at low adherence levels. As our analysis adopted a workflow similar to Zhang et al, our model may also predict comparingly high effectiveness at low adherence of F/TDF. As indicated above, if counterfactual background incidences were used (as opposed to estimating incidence by dichotomizing observation time) our mechanistic model may overpredict the prophylactic efficacy of F/TAF.

The clinical trial simulation results are sensitive to the background incidence rate selected. Using the dichotomization method, the background incidences were estimated as 3.30 (95% CI: 2.3, 4.4) and 2.20 (95% CI: 1.3, 3.4) per 100 person‐years for the F/TAF and F/TDF arms, respectively. The higher incidence rate in F/TAF arm could partially explain the underestimated total number of infections in our simulation. However, differences across treatment arms are difficult to interpret in a randomized trial. Therefore, we adopted the background incidence rate reported by Bekker et al, derived from the cross‐sectional incidence cohort.

There are several limitations in our analysis. First, our simulated PBMC TFV‐DP concentrations during pregnancy differ from those reported by Mugwanya^21^ and Joseph Davey.^20^ Those two studies reported no significant reduction in PBMC TFV‐DP with F/TDF during pregnancy. This discrepancy reflects our PK model assumptions linking PBMC anabolite concentrations to plasma TFV concentrations. Second, we assumed that concentrations in PBMCs are the best surrogate to predict prevention efficacy and did not evaluate vaginal and rectal tissue concentrations. Since our results suggest that PBMCs may not be considered as the only reliable variables for predicting the effectiveness of oral PrEP in women, future pharmacological research and computational modeling combining both PBMC and vaginal and rectal tissue exposure may address this issue. A mechanism‐based PK model that can simultaneously describe TDF, TAF, and FTC and their active anabolite in peripheral matrices and local tissue may be helpful to further evaluate the contribution from each component. Lastly, unlike TDF, there is currently no in vitro study evaluating the synergistic effect between TAF and FTC. Our assumption that TAF and FTC exhibit similar synergy to F/TDF may not accurately reflect the true biological interaction.

## Author Contributions

Rachel K. Scott, Yifan Yu, Jeremiah D. Momper, Robert Bies, and Craig W. Hendrix designed the research. Yifan Yu performed the research. Yifan Yu analyzed the data. Max von Kleist and Lanxin Zhang contributed new analytical tools. All authors wrote and reviewed the manuscript.

## Conflicts of Interest

Renee Heffron serves as a consultant to Merck, Sharp & Dohme and has been a Principal Investigator for studies that received donated FTC/TDF medication from Gilead Sciences. Robert Bies serves as a consultant for Advanced Bioscience Laboratories. Craig W. Hendrix holds several patents and receives royalties related to PrEP topical microbicides, and is a founder of Prionde Biopharma, LLC, a PrEP microbicide company; all conflicts are managed by Johns Hopkins University and the HPTN. Dvora L. Joseph Davey receives research support from ViiV Healthcare, Merck, and Gilead Sciences. Kristina M. Brooks previously received consulting fees from ViiV Healthcare. Peter L. Anderson receives research contracts from Gilead Sciences, with payments made to his institution. Mark Mirochnick receives research support through his institution from ViiV Healthcare and Merck. Rachel K. Scott has participated in advisory boards and received investigator‐sponsored research funding from ViiV Healthcare and Gilead Sciences, managed by her institution. Yifan Yu, Gustavo F. Doncel, Brookie M. Best, Mark A. Marzinke, Landon Myer, Connie Celum, Jenell Coleman, Lanxin Zhang, and Max von Kleist report no conflicts of interest.

## Funding

Research reported in this publication was supported by Eunice Kennedy Shriver National Institute of Child Health and Human Development (NICHD) of the National Institutes of Health under award number R21HD106582. Additional support was provided by the NICHD Maternal and Pediatric Precision in Therapeutics (MPRINT) under grant number P30HD106451. The content is solely the responsibility of the authors and does not necessarily represent the official views of the National Institutes.

## Supporting information



Supporting Information

## Data Availability

The data that support the findings of this study are available on request from the corresponding author. The data are not publicly available due to privacy or ethical restrictions.
