# Supplementary material for: Predicting Oral HIV Pre‐Exposure Prophylaxis Efficacy in Cisgender Women Across Pregnancy and Postpartum
Source: J Clin Pharmacol. 2026 Sep 3;66(9):e70278. doi: 10.1002/jcph.70278 (PMC13539293; doi:10.1002/jcph.70278)
Supplement: Supplementary file 1 [file JCPH-66-0-s001.docx]

**Supplementary Online Material**

**Predicting Oral HIV Pre-Exposure Prophylaxis Efficacy in Cisgender Women across Pregnancy and Postpartum**

Yifan Yu MS^1^, Kristina M. Brooks PharmD^2^, Gustavo F. Doncel MD^3^, Brookie M. Best PharmD^4,5^, Mark A. Marzinke PhD^6^, Mark Mirochnick MD^7^, Peter L. Anderson PharmD^2^, Landon Myer MD^8^, Connie Celum MD^9^, Renee Heffron PhD^10^, Jenell Coleman MD^6^, Dvora L. Joseph Davey PhD^8,11^, Lanxin Zhang PhD^12^, Max von Kleist PhD^12^, Craig W. Hendrix MD^6^, Jeremiah D. Momper PharmD, PhD^4^, Robert Bies PharmD, PhD^1^, Rachel K. Scott MD^13,14,§^

1. University at Buffalo, School of Pharmacy and Pharmaceutical Sciences, Buffalo, NY, USA.

2. Skaggs School of Pharmacy and Pharmaceutical Sciences, University of Colorado-Anschutz Medical Campus, Aurora, CO, USA.

3. CONRAD, Eastern Virginia Medical School, Old Dominion University, Norfolk, VA, USA.

4. University of California San Diego, Skaggs School of Pharmacy and Pharmaceutical Sciences, La Jolla, CA, USA.

5. Pediatrics Department, University of California San Diego School of Medicine-Rady Children’s Hospital San Diego, San Diego, CA, USA.

6. School of Medicine, Johns Hopkins University, Baltimore, MD, USA.

7. Boston University Chobanian & Avedisian School of Medicine, Boston, MA, USA.

8. Division of Epidemiology and Biostatistics, School of Public Health, University of Cape Town, Cape Town, South Africa.

9. Departments of Global Health, Medicine & Epidemiology, University of Washington, Seattle, WA, USA.

10. Department of Medicine, University of Alabama at Birmingham, Birmingham, AL, USA.

11. Division of Infectious Diseases, Geffen School of Medicine, University of California Los Angeles, Los Angeles, CA, USA.

12. Project group 5 “Systems medicine of Infectious Disease”, Robert-Koch Institute, Berlin, Germany

13. Medstar Health, Washington D.C. USA

14. Georgetown University School of Medicine, Washington D.C. USA

^§^ **Corresponding author**: Rachel K. Scott

MedStar Health Research Institute & MedStar Washington Hospital Center

110 Irving Street NW, East Building 5108,

Washinton, DC, 20010, US

Phone number: 202-877-7473

Email: Rachel.K.Scott@Medstar.net

**FTC population PK model**

The ODEs of the final FTC PK model are provided below:

$\frac{dX_{1}}{dt}=-{Ka}_{1}X_{1}$; Extravascular (oral) dosage compartment FTC

$\frac{dX_{2}}{dt}={Ka}_{1}X_{1} -\frac{CL}{V_{2}}X_{2}+\frac{Q_{1}}{V_{3}}X_{3}-\frac{Q_{1}}{V_{2}}X_{2}+\frac{Q_{2}}{V_{4}}X_{4}-\frac{Q_{2}}{V_{2}}X_{2}$; Central compartment FTC

$\frac{dX_{3}}{dt}=\frac{Q_{1}}{V_{2}}X_{2}-\frac{Q_{1}}{V_{3}}X_{3}$; First peripheral compartment FTC

$\frac{dX_{4}}{dt}=\frac{Q_{2}}{V_{2}}X_{2}-\frac{Q_{2}}{V_{4}}X_{4}$; Second peripheral compartment FTC

$\frac{dC_{PBMC}}{dt}=\frac{V_{max}}{K_{m}+C_{2}}C_{2}- K_{el-PBMC}C_{PBMC}$; PBMCs FTC-TP

The parameters final estimates are summarized in Tables S1.

**Table S1:** Final estimates of FTC PK parameters.

| Parameter | Final Estimate (RSE%) | BSV %CV (RSE%) |
| --- | --- | --- |
| CL/F (L/h) | 16.7 (11%) | 32.1% (10%) |
| V2/F (L) | 62.2 (16%) | 21.3% (25%) |
| KA (/h) | 1.24 (21%) | 39.6% (24%) |
| Q1/F (L/h) | 4.58 (56%) |  |
| V3/F (L) | 18.1 (33%) |  |
| Q2/F (L/h) | 2.63 (13%) |  |
| V4/F (L) | 80.8 (4%) |  |
| $V_{\max}$ (µmol/L/h) | 5.54 (6%) | 22.6% (23%) |
| $k_{m}$ (µmol/L) | 0.273 (13%) | 53.9% (18%) |
| $k_{el-PBMC}$ (/h) | 0.356 (8%) | 27.4% (20%) |
| PROP (FTC) | 39.9% (3%) |  |
| PROP (FTC-TP) | 38.0% (3%) |  |

**Abbreviations: %RSE, relative standard error; BSV, between subject variability; %CV, coefficient of variation in percentage terms; CL/F, plasma FTC apparent clearance; V2/F, FTC central compartment apparent volume of distribution; KA1, FTC absorption rate constant; Q1/F, plasma FTC apparent intercompartmental clearance between central compartment and the first peripheral compartment; V3/F, FTC first peripheral compartment apparent volume of distribution; Q2/F, plasma FTC apparent intercompartmental clearance between central compartment and the second peripheral compartment; V4/F, FTC second peripheral compartment apparent volume of distribution;** $V_{\max}$**, maximum reaction velocity;** $K_{m}$**, Michaelis constant;** $k_{el-PBMC}$**, elimination rate from PBMC; PROP, proportional error.**

**Combined effect of TFV-DP and FTC-TP**

**Figure S1:** Combined effect of TFV-DP and FTC-TP based on the MMOA model developed by Iannuzzi and von Kleist. The x and y axes represent the log10 PBMC FTC-TP and TFV-DP concentrations, respectively. Color shading indicates η, the inhibition of the cell infection. Panel A shows the simulated combination effect accounting for dNTP depletion. Panel B shows the simulated effect assuming no synergistic interaction between two drugs.

**Dichotomization of the PURPOSE 1 trail results**

In brief, we first calculated the proportion of participants with adherence less than 2 pills per week, derived from the total number of DBS samples analyzed in the PURPOSE 1 trial (N=849 for F/TAF and 439 for F/TDF). We then assumed that participants in the low-adherence group receive no drug at the time of viral exposure. Based on this assumption, we divided the total number of participants, total follow-up time, and total number of infections into “no drug” group and “some drug” group. Finally, the background incidences were sampled using the cumulative distribution function reported by Zhang et al.

Table S2 shows the dichotomization results. Figure S1 shows the distribution of background HIV incidence sampled from the fitted gamma distribution (mean = 2.41 per 100 person-years, SE = 0.35), and from the dichotomization method. The mean background incidence rates estimated using the dichotomization method were 3.30 (95% CI: 2.3, 4.4) per 100 person-years for F/TAF arm and 2.20 (95% CI: 1.3, 3.4) per 100 person-years for the F/TDF arm.

We simulated the “no drug” group in both F/TAF and F/TDF arms (see method) to validate the different background incidence rates. The results were summarized in Tables S3.

Adherence levels for the PURPOSE 1 trail were estimated based on the reported dried blood spot data (N=849 for the F/TAF group and N=439 for the F/TDF group). For the three adherence categories (low, medium, and high) used in PURPOSE 1 trial, we assumed that the probability of taking any given number of pills within each category was equal, with the exception that participants in the low-adherence group were assumed to take no drug. This assumption is consistent with the dichotomization approach applied in the analysis presented here. The predicted adherence levels for the PURPOSE 1 trial are summarized in Figure S2.

Based on these predicted adherence levels, the average prophylactic efficacy in the PURPOSE 1 trial was estimated to be 41.1% in the F/TAF arm and 22.2% in the F/TDF arm. The simulation results based on the background incidence estimated using the dichotomization method are shown in Figure S3.

**Table S2:** Dichotomization results

| Study arm | Total observation time (person-years) | Total number of participants | Total number of infections | Observation time in “no drug” group | Number of participants in “no drug” group | Number of infections in “no drug” group |
| --- | --- | --- | --- | --- | --- | --- |
| PURPOSE 1 F/TAF arm | 1930 | 2137 | 39 | 1119 | 1240 | 36 |
| PURPOSE 1 F/TDF arm | 947 | 1070 | 16 | 720 | 813 | 15 |

**Table S3:** Simulation results for no drug group

| Study arm | Observed number of infections in “no drug” group | Simulated number of infections in “no drug” group using the incidence rate from dichotomization (95% prediction interval) | Simulated number of infections in “no drug” group using the reported incidence rate (95% prediction interval) |
| --- | --- | --- | --- |
| PURPOSE 1 F/TAF arm | 36 | 37 (21, 55) | 26 (15, 41) |
| PURPOSE 1 F/TDF arm | 15 | 16 (7, 28) | 17 (9, 27) |

**Figure S2**: Error bars show the mean and 95% quantile range of 1000 sampled background incidences. The reported background incidence was derived from the cross-sectional incidence cohort, sampled from $r_{inf-background}$～Gamma (α=47.52, β=19.72). The background incidences in F/TAF and F/TDF arms were estimated using the dichotomization method described in the method section. Confidence intervals were estimated using Wilson’s method.

**Figure S3**: The estimated adherence level based on all the DBS TFV-DP concentrations analyzed in the PURPOSE 1 trial. Based on these predicted adherence levels, the average prophylactic efficacy in the PURPOSE 1 trial was estimated to be 41.1% in the F/TAF arm and 22.2% in the F/TDF arm.

**Clinical Trial Simulation**

**Figure S4:** Clinical trial simulation results with the background incidence derived from the cross-sectional incidence cohort. A and C, simulated cumulative number of HIV infections over time in the F/TAF arm and F/TDF arm across 1000 replicates. The red dashed line indicated the observed HIV infections in the PURPOSE 1 study. B and D, Distribution of total simulated HIV infections in the F/TAF arm and F/TDF arm.

**Figure S5:** Clinical trial simulation results with the background incidence estimated using dichotomization method. A and C, simulated cumulative number of HIV infections over time in the F/TAF arm and F/TDF arm across 1000 replicates. The red dashed line indicated the observed HIV infections in the PURPOSE 1 study. B and D, Distribution of total simulated HIV infections in the F/TAF arm and F/TDF arm. Across simulations, there were a median of 37 infections and a 95% quantile range of 22 to 55 in the F/TAF arm; there were a median of 16 infections and a 95% quantile range of 7 to 30 in the F/TDF arm.

**Predicted Minimal Prophylactic Efficacy**

We performed additional simulation using different adherence patterns with even dosing interval for 6 weeks and calculated the median and 90% simulation interval of minimal predicted efficacy.

**Table S4:** Predicted minimal efficacy across different adherence and reproductive stages

| Adherence | Predicted minimal efficacy | | | |
| --- | --- | --- | --- | --- |
|  | F/TDF nonpregnant | F/TDF 2^nd^ Trimester | F/TAF nonpregnant | F/TAF 2^nd^ Trimester |
| 7 pills/week | 100% (97.9%, 100%) | 99.9% (96.6%, 100%) | 100% (99.9%, 100%) | 100% (99.6%, 100%) |
| 6 pills/week | 99.9% (96.7%, 100%) | 99.6% (94.8%, 100%) | 100% (99.7%, 100%) | 100% (99.2%, 100%) |
| 5 pills/week | 99.6% (94.6%, 100%) | 99.1% (91.6%, 100%) | 100% (99.1%, 100%) | 100% (98.2%, 100%) |
| 4 pills/week | 98.9% (90.8%, 100%) | 97.7% (86.3%, 100%) | 100% (97.9%, 100%) | 99.8% (96.2%, 100%) |
| 3 pills/week | 96.9% (83.6%, 99.9%) | 94.5% (77.2%, 99.3%) | 99.5% (94.7%, 100%) | 98.7% (91.8%, 100%) |
| 2 pills/week | 89.9% (67.7%, 99.6%) | 84.5% (59.3%, 98.9%) | 96.6% (85.0%, 99.9%) | 94.1% (79.9%, 99.7%) |
| 1 pill/week | 50.9% (26.3%, 85.7%) | 41.9% (20.4%, 77.7%) | 72.2% (43.9%, 92.9%) | 65.0% (37.0%, 88.5%) |
| 1 pill/2 weeks | 10.3% (3.6%, 26.6%) | 7.7% (2.7%, 20.9%) | 21.6% (7.4 %, 50.1%) | 17.6% (5.8%, 44.6%) |
